# Understanding diagnosis and management of dementia and guideline implementation in general practice: a qualitative study using the theoretical domains framework

**DOI:** 10.1186/1748-5908-9-31

**Published:** 2014-03-03

**Authors:** Kerry Murphy, Denise A O’Connor, Colette J Browning, Simon D French, Susan Michie, Jill J Francis, Grant M Russell, Barbara Workman, Leon Flicker, Martin P Eccles, Sally E Green

**Affiliations:** 1 School of Public Health and Preventive Medicine, Monash University, 99 Commercial Road, Melbourne, VIC 3004, Australia; 2 School of Primary Health Care, Monash University, 270 Ferntree Gully Road, Notting Hill, VIC 3168, Australia; 3 Centre for Health, Exercise and Sports Medicine, Melbourne School of Health Sciences, University of Melbourne, 200 Berkeley St, Carlton, VIC 3010, Australia; 4 Department of Psychology, University College London, London, UK; 5 School of Health Sciences, City University London, London, UK; 6 Southern Academic Primary Care Research Unit, School of Primary Health Care, Monash University, 270 Ferntree Gully Road, Notting Hill, VIC 3168, Australia; 7 Department of Medicine, Monash Medical Centre and Monash University, Monash VIC 3800, Australia; 8 Western Australian Centre for Health & Ageing, Centre for Medical Research, University of Western Australia, 35 Stirling Highway, Crawley, WA 6009, Australia; 9 Institute of Health and Society, Newcastle University, Baddiley-Clark Building, Richardson Road, Newcastle upon Tyne, UK

**Keywords:** Dementia, General practitioners (GPs), Cognitive assessment, Depression assessment, Theoretical Domains Framework (TDF), Guideline implementation

## Abstract

**Background:**

Dementia is a growing problem, causing substantial burden for patients, their families, and society. General practitioners (GPs) play an important role in diagnosing and managing dementia; however, there are gaps between recommended and current practice. The aim of this study was to explore GPs’ reported practice in diagnosing and managing dementia and to describe, in theoretical terms, the proposed explanations for practice that was and was not consistent with evidence-based guidelines.

**Methods:**

Semi-structured interviews were conducted with GPs in Victoria, Australia. The Theoretical Domains Framework (TDF) guided data collection and analysis. Interviews explored the factors hindering and enabling achievement of 13 recommended behaviours. Data were analysed using content and thematic analysis. This paper presents an in-depth description of the factors influencing two behaviours, assessing co-morbid depression using a validated tool, and conducting a formal cognitive assessment using a validated scale.

**Results:**

A total of 30 GPs were interviewed. Most GPs reported that they did not assess for co-morbid depression using a validated tool as per recommended guidance. Barriers included the belief that depression can be adequately assessed using general clinical indicators and that validated tools provide little additional information (theoretical domain of ‘Beliefs about consequences’); discomfort in using validated tools (‘Emotion’), possibly due to limited training and confidence (‘Skills’; ‘Beliefs about capabilities’); limited awareness of the need for, and forgetting to conduct, a depression assessment (‘Knowledge’; ‘Memory, attention and decision processes’). Most reported practising in a manner consistent with the recommendation that a formal cognitive assessment using a validated scale be undertaken. Key factors enabling this were having an awareness of the need to conduct a cognitive assessment (‘Knowledge’); possessing the necessary skills and confidence (‘Skills’; ‘Beliefs about capabilities’); and having adequate time and resources (‘Environmental context and resources’).

**Conclusions:**

This is the first study to our knowledge to use a theoretical approach to investigate the barriers and enablers to guideline-recommended diagnosis and management of dementia in general practice. It has identified key factors likely to explain GPs’ uptake of the guidelines. The results have informed the design of an intervention aimed at supporting practice change in line with dementia guidelines, which is currently being evaluated in a cluster randomised trial.

## Background

Dementia is an increasingly prevalent, global problem that results in substantial health, social and financial consequences for the individuals affected, their caregivers, and society [1]. The worldwide estimate of the number of people with dementia in 2010 was 35.6 million, and this is projected to increase to 115 million by 2050 [2]. In Australia, there are approximately 298,000 people living with dementia and this is expected to reach 900,000 by 2050 [3].

General practitioners (GPs) play an important role in the detection and management of dementia. They are generally the first point of contact for patients with suspected cognitive impairment or dementia, and they are often primarily responsible for the ongoing management of the patient once a diagnosis has been confirmed [4-7]. Early detection of symptoms can allow reversible causes of cognitive decline to be addressed and co-morbidities, such as depression, to be identified and managed. Early diagnosis of dementia enables access to education, support and counselling services to assist patients and carers, and also provides patients and carers with the opportunity and time to plan for the future and organise their personal and financial affairs [8-11]. Early access to dementia-modifying medication, such as acetyl-cholinesterase inhibitors, can produce symptomatic benefits for some patients and may result in an increase in functionality [8-10,12].

Evidence-based guidelines for the detection, diagnosis and management of dementia relevant to primary care, such as those published by the Scottish Intercollegiate Guidelines Network (SIGN) [13], are accessible to practitioners through various sources including guideline clearinghouses and guideline development organisation websites. These guidelines include a series of evidence-based recommendations for investigations and interventions which, if followed, optimise the health outcomes for people with dementia. The SIGN-recommended investigations and interventions include: conducting a formal cognitive assessment using the Mini-Mental State Examination (MMSE) in individuals with suspected cognitive impairment; assessing for co-morbid depression using a validated tool; use of structural imaging (*e.g*., brain CT) in the diagnostic workup of patients with suspected dementia; access to dementia-modifying medication in patients with confirmed dementia; provision of caregiver support and training; and promotion of cognitive stimulation and recreational activities. A systematic search of dementia guidelines published after the SIGN guideline conducted by our team identified 14 guidelines, the majority of which share the same recommendations as SIGN.

Despite availability and dissemination of guidelines for diagnosis and management of dementia, gaps between recommended and current practice still exist [14-20]. For example, a study of dementia care in the US found that concordance with a number of dementia care process measures (drawn from guideline recommendations) ranged from 9% to 79%, with concordance for 11 of these processes being less than 40% [15]. Formal cognitive assessment may not be conducted in as many as 30% to 50% of cases [15,18]. Similar findings have been reported in relation to assessment for co-morbid depression in patients being evaluated for dementia [15,16,18]. Data from Australian studies suggests that formal cognitive testing is not conducted to the extent recommended [14,20].

A systematic review of barriers to physicians’ use of guidelines has identified a number of factors that may influence uptake, including lack of awareness or familiarity with guidelines, lack of agreement, lack of self-efficacy, lack of outcome expectancy, the inertia of previous practice, and external barriers to guideline use [21]. A systematic review specifically focussing on barriers to recommended diagnosis and management of dementia in primary care has also identified several influences on primary care practitioners’ practice [22]. These include lack of support, time constraints, financial constraints, stigma associated with diagnosis, diagnostic uncertainty, insufficient knowledge or experience, and difficulties disclosing the diagnosis. While these go some way to explaining the practice gap, none of the studies used theory to investigate the causes of implementation difficulties. Use of theory has been advocated to facilitate a comprehensive assessment of mediators of behaviour change for designing interventions that are most likely to bring about behaviour change [23,24]. Additionally, few studies in the systematic review explored the causes of implementation difficulties specifically in relation to individual recommendations described in behavioural terms (*i.e*., who needs to do what, how and where); this may have limited the accuracy and/or specificity of the barriers identified.

Theory provides an explicit statement of the structural and psychological processes that are hypothesised to influence behaviour and, as such, is useful for investigating implementation difficulties, informing the design of practice change interventions and contributing to the evidence base on which to select interventions [23-25]. Theory can provide information about the mechanisms involved in clinician behaviour change, and these mechanisms can be systematically investigated and targeted with behaviour change techniques and components to bring about change. If assessments of implementation difficulties are not undertaken within a theoretical approach, then the resulting interventions are likely to be limited to pragmatic rather than generalisable solutions, and opportunities to investigate the mediating pathways of behaviour change and optimise interventions will be limited [26].

Many theories are available to guide the assessment of implementation difficulties and for understanding behaviour and behaviour change [27]. In an effort to make theory more accessible to those interested in implementation and behaviour change, the Theoretical Domains Framework (TDF) was developed by a team of psychology theorists in collaboration with implementation researchers. The TDF represents an integrated theoretical framework of a number of domains and theoretical constructs synthesised from 33 theories and 128 constructs, which can be used to identify theoretical explanations for implementation difficulties and to inform the design of implementation interventions [28]. The TDF includes 12 theoretical domains: ‘Knowledge’, ’Skills’, ‘Social/professional role and identity’, ‘Beliefs about capabilities’, ‘Beliefs about consequences’, ‘Motivation and goals’, ‘Memory, attention and decision processes’, ‘Environmental context and resources’, ‘Social influences’, ‘Emotion’, ‘Behavioural regulation’ and ‘Nature of the behaviours.’ Since this study was completed, the TDF has been independently validated to confirm the optimal domain structure, content and labels of the framework [29]. To date, a number of empirical studies have used the TDF to explore implementation problems in different clinical areas [30], including low back pain [26,31-33], hand hygiene [34,35], blood transfusion [36,37], medication prescribing [38], and schizophrenia [39] but not dementia.

The aim of this study was to use the TDF to explore GPs’ reported practice in relation to the diagnosis and management of dementia and to describe, in theoretical terms, the proposed explanations for practice that was and was not consistent with recommended guidance. The study is the first phase of a larger cluster randomised trial testing the effectiveness of a theory-informed intervention to increase GPs’ uptake of a dementia guideline in general practice, ‘Investigating Research Implementation Strategies for the care of older adults with suspected cognitive impairment’ (IRIS) [40]. This paper describes reported practices and theoretical explanations in relation to two key diagnostic recommendations of the SIGN guideline (with level B evidence) for which there are identified evidence-practice gaps [13]. These practices constitute primary targets for behaviour change in the IRIS intervention. The SIGN recommendations are: conducting a formal cognitive assessment using the MMSE in individuals with suspected cognitive impairment, and assessing for co-morbid depression using a validated tool. The results of the current qualitative study have been used to design the IRIS intervention by targeting the identified barriers and enablers with inclusion of relevant behaviour change techniques. A description of the development and content of the IRIS intervention and the subsequent cluster randomised trial findings will be reported separately.

## Methods

### Design

Qualitative study using semi-structured interviews based on the TDF.

### Participants and setting

GPs managing people with suspected cognitive impairment or dementia and practising in the Australian state of Victoria. Suspected cognitive impairment or dementia was defined by the GPs.

### Procedure

The contact details of a random sample of GPs practising in the Australian state of Victoria, sampled equally from two geographical strata (metropolitan, regional/rural), was supplied by the Australian Medical Publishing Co (AMPCo), a subsidiary of the Australian Medical Association, which provides lists of Australian medical practitioners. An invitation to participate (including explanatory statement and consent form) was mailed, in batches of 100, to 700 GPs in the sampling frame. GPs currently managing people with suspected cognitive impairment or dementia and wishing to participate opted in to the study by returning a signed consent form. Data collection commenced as soon as GPs opted into the study, and analysis occurred after each interview. Recruitment continued to the point where data saturation was achieved and no new information was forthcoming.

Participants took part in a single, one-hour interview with an experienced qualitative researcher (KM), either face-to-face in their practice rooms or by telephone. An interview guide with questions and prompts informed by the TDF guided the discussion. The interviews focused on the diagnosis and management recommendations from the SIGN guideline, with some adaptation for the Australian context, together with recommendations that were considered best practice by the IRIS clinical investigators (Table 1). The interviews were audio-taped and transcribed verbatim. Transcriptions were cross-checked against the audio recordings for accuracy and de-identified. Participants were provided with a copy of their interview transcription and invited to check it for accuracy and return any amendments. Participants were offered an honorarium to cover the costs associated with their participation and could claim Continuing Professional Development points for their participation.

**Table 1:** Diagnosis and management recommendations investigated

**Recommendation**	**Details and source**
Conduct a formal cognitive assessment using a validated scale (*e.g*., MMSE) in individuals with suspected cognitive impairment.	SIGN guideline recommends the MMSE should be used for cognitive testing of individuals with suspected cognitive impairment (Grade B recommendation).
Assess for co-morbid depression using a validated tool (*e.g*., Geriatric Depression Scale or others).	SIGN guideline recommends considering the presence of co-morbid depression as part of the assessment for suspected dementia (Grade B recommendation). Evidence underpinning the guideline advocates use of validated tools for assessing depression (*e.g*., Geriatric Depression Scale).
Refer for pathology testing.	This is a SIGN guideline good practice point. Supported by other guidelines and considered best practice by the IRIS clinical investigators to facilitate exclusion of potentially reversible causes of dementia.*
Refer for head/brain computed tomography (CT) scan.	SIGN guideline recommends structural imaging should form part of the diagnostic work up of patients with suspected dementia (Grade C recommendation). We focus only on referral for CT scan since GPs in Australia cannot refer for a Medicare rebatable magnetic resonance imaging (MRI).
Review current medication (prescription and over the counter) that may cause cognitive impairment.	Not a recommendation of the SIGN guideline. Supported by other guidelines and considered best practice by the IRIS clinical investigators to eliminate possible other causes of dementia-like symptoms.*
Disclose or reinforce a diagnosis of dementia.	Not a recommendation of the SIGN guideline. The SIGN guideline recommends that healthcare professionals should be aware that many people with dementia can understand their diagnosis, receive information, and be involved in decision making (Grade C recommendation); that some people with dementia may not wish to know their diagnosis (Grade C recommendation); and that in some situations disclosure of a diagnosis of dementia may be inappropriate (Grade D recommendation). Supported by other guidelines and considered best practice by the IRIS clinical investigators.*
Refer to specialist (including via Cognitive, Dementia and Memory Service [CDAMS]) for access to dementia-modifying medications.	SIGN guideline makes recommendations about specific pharmacological interventions (*e.g*., use of cholinesterase inhibitors) (Grade B recommendations). Access to dementia-modifying medication is via specialist referral in Australia.
Provide information on, or refer for, recreational activities.	SIGN guideline recommends that recreational activities should be encouraged to enhance the quality of life and well-being of people with dementia (Grade B recommendation). Recreational activities may include current or previous interests or the introduction of new recreational activities. Alzheimer’s Australia offer education and training and facilitate support groups for people with dementia and their carers.
Provide information on, or refer for, activities to promote cognitive stimulation.	SIGN guideline recommends that cognitive stimulation should be offered to people with dementia (Grade B recommendation). Cognitive stimulation may occur through participation in recreational activities, via support from a carer or through formal cognitive stimulation activities. Alzheimer’s Australia offer education and training and facilitate support groups for people with dementia and their carers.
Provide, or refer for, caregiver training.	SIGN guideline recommends that caregivers should receive training on interventions that are effective for people with dementia (Grade B recommendation). Alzheimer’s Australia offer education and training and facilitate support groups for people with dementia and their carers.
Give advice re. respite care.	Not a recommendation of the SIGN guideline. Supported by other guidelines and considered best practice by the IRIS clinical investigators.*
Promote awareness of changing driving capacity as disease progresses.	Not a recommendation of the SIGN guideline. Supported by other guidelines and considered best practice by the IRIS clinical investigators.*
Discussion of legal issues.	Not a recommendation of the SIGN guideline. Supported by other guidelines and considered best practice by the IRIS clinical investigators.*

*Recommendations considered best practice by the IRIS clinical investigators, arrived at through discussion and consensus.

### Interview guide

The interview guide (see Additional file 1) covered the 13 recommended behaviours outlined in Table 1. GPs were first asked general questions about how they became aware of patients with suspected cognitive impairment or dementia and what steps they then took. They were also asked about each of the recommended behaviours to gain insight into their reported practice and the factors that hindered or enabled achievement of the behaviours. The interview guide was based on the TDF and was developed by a multi-disciplinary team, including investigators with clinical expertise in dementia management and those with expertise in behaviour change and implementation research. The interview guide was piloted with three GPs prior to data collection to assess its comprehensiveness, practicability and acceptability.

### Analysis

**Figure 1:**
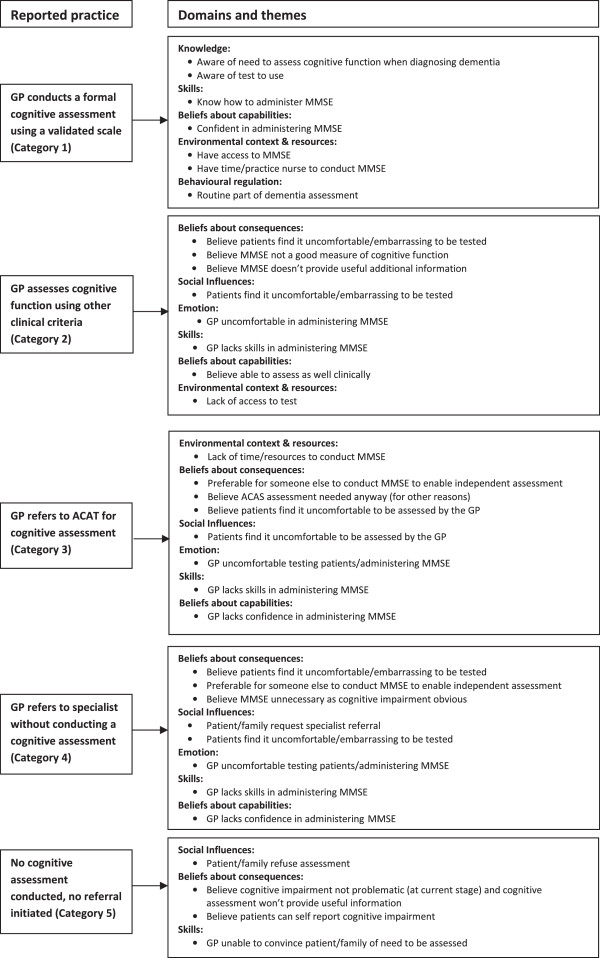
**Nature of reported practice and factors perceived to influence practice for recommended behaviour 1: conduct a formal cognitive assessment using a validated scale (*e.g*., MMSE).**

The interview transcripts were analysed using content analysis after each interview, and both the manifest and latent content was examined [41-43]. First, the nature of participants’ current practice in relation to each of the recommended behaviours was identified, and the data were categorised on the basis of the identified groupings (Figure 1). For example, data from GPs reporting they conducted a MMSE or similar validated assessment with patients they suspected of having cognitive impairment were coded alike based on their reported practice. The data for each grouping were then examined to look for consistencies and variations to ascertain whether it could be classified further. Next, the data in each grouping were content analysed to identify the factors perceived to influence the achievement of the recommended behaviours (*i.e*., barriers and enablers). These factors were then thematically mapped to the domains of the TDF to enable identification of possible theoretical explanations for practice that was and was not consistent with recommended guidance. Metropolitan and regional/rural interviews were analysed separately to investigate geographic variations.

The development of the coding frame and initial analysis of interview data was undertaken by the researcher who conducted the interviews, and a random subset of 20% of interviews was independently coded and analysed by a second researcher with expertise in qualitative methods as a method of verification of the initial analysis. The thematic analysis of the factors to the TDF was conducted by the researcher who conducted the interviews and checked by a second researcher with expertise in the TDF. Coding by the independent researchers was compared manually, and any discrepancies were discussed until agreement was reached. Few discrepancies between coders were identified.

### Ethics

This study was approved by the Monash University Human Research Ethics Committee (MUHREC) - Project Number: CF08/1291 – 2008000627.

## Results

### Participants

A total of 30 GPs (18 male, 12 female) who reported that they manage people with suspected cognitive impairment or dementia from 30 general practices throughout Victoria, Australia (13 metropolitan, 17 regional/rural), participated in the study (4% of GPs invited). Participants varied in terms of years since graduation from medical training (mean 28.00 years, SD 8.95 years), practice size (11 solo, 19 group; median practice size 2, range 1 to 11), and number of patients in their care with a confirmed diagnosis of dementia (ranging from 0 to 25). In total, 24 interviews were conducted face-to-face.

### Conducting a formal cognitive assessment using a validated scale (*e.g*., MMSE) (recommended behaviour 1)

This study identified a range of clinical behaviours that GPs engage in relating to the conduct of a formal cognitive assessment in patients with suspected cognitive impairment. These were classified into five categories (Figure 1):

Category 1. GP conducts a formal cognitive assessment using a validated scale (MMSE).

Category 2. GP assesses cognitive function by other clinical methods (not using a validated tool).

Category 3. GP refers the patient to a specialised Aged Care Assessment Service (ACAS) for a cognitive assessment (*i.e*., GP does not conduct a cognitive assessment) [ACAS comprises a team of health professionals who conduct assessments of older people to assist them to gain access to services most appropriate to meet their care needs. ACAS is publically funded in Australia].

Category 4. GP refers the patient to a specialist for assessment (*i.e*., GP does not conduct a cognitive assessment).

Category 5. No cognitive assessment is conducted, and no referral to another health professional or service for this assessment is initiated by the GP.

For the vast majority of GPs in this study, assessing the cognitive function of their patients either themselves or via their practice nurse was their normal procedure. In almost all cases, this was done using a MMSE, or similar validated scale (Category 1), rather than assessing the patient using other clinical criteria (Category 2). In contrast, referring a patient to ACAS or a specialist for assessment (Categories 3 and 4) was a situation-specific practice, in that it occurred with some patients in certain situations and was not undertaken in all situations by GPs who described doing this. In different circumstances, or with different patients, the GP may conduct the assessment themselves. However, a small number of the GPs preferred to routinely have their patients assessed by a third party (ACAS, specialist) for various reasons (discussed below) (Category 3 and 4). Not conducting a cognitive assessment (Category 5) occurred when a GP was unable, or decided not, to assess a patient’s cognitive function at a certain time, although they may do so at a later time. Again, this practice was generally situation-specific.

The factors perceived to influence the behaviour of GPs in each category are described below and summarised in Figure 1. The barriers and enablers that were elicited through the interviews and identified via content analysis were then thematically mapped to the relevant theoretical domains of the TDF [28]. Illustrative quotes from participants are provided where relevant.

### Factors perceived to influence practice, mapped to the TDF

#### Category 1. GP conducts a formal cognitive assessment using a validated scale (MMSE)

The main factors enabling the conduct of a formal cognitive assessment using the MMSE were an awareness of the need to assess cognitive function in patients in whom cognitive impairment or dementia is suspected – combined with an awareness of the appropriate test to use (Knowledge), being able to access the test (Environmental context and resources), and knowing how to and being confident in administering it (Skills; Beliefs about capabilities). Another important factor was having the time needed, or the availability of a practice nurse, to conduct the test (Environmental context and resources).

The advantages of a practice nurse are apparent in the comment made by one GP: ‘If I feel anything is serious I say, “look let’s do a formal memory test for you” … and we have the facility of a practice nurse who can go out and do home health assessments, and they usually do a formal Mini Mental test on the patient’ (GP 20 – Metro).

For most of these GPs the conduct of a cognitive assessment was a routine part of a dementia assessment process (Behavioural regulation), as the following comment indicates: ‘Okay, well the first thing I’d do is do a Mini Mental’ (GP 12 – Metro).

#### Category 2. GP assesses cognitive function through use of other clinical criteria

A common reason cited for preferring to assess patients through use of other clinical criteria rather than using a validated scale was a belief that patients found responding to validated cognitive assessments, such as the MMSE, embarrassing or uncomfortable (Beliefs about consequences; Social Influences). As one GP explained: ‘Oh, they just get embarrassed, and the questions are a bit demeaning, I think. If I was asked those questions, I’d be a bit insulted’ (GP 13 – Rural).

Other reasons were that they either believed that a MMSE was not a good measure of cognitive function (particularly with patients from non-English speaking backgrounds) (Beliefs about consequences), or that they believed they could assess cognitive function through use of other clinical criteria (Beliefs about capabilities for performing a competing behaviour) and that a MMSE did not necessarily provide additional useful information (Beliefs about consequences). The latter view is reflected in the following statement: ‘Oh sometimes I do an MMSE. … I don’t find it that useful. I mean … from conversation you can really find which way they’re going. I mean… so the MMSE is … it doesn’t give me additional information I find’ (GP 25 – Metro).

Another barrier to the use of a validated tool when conducting a cognitive assessment was the discomfort some GPs reported experiencing when using such a tool with patients (Emotion), possibly due to lack of training or experience in its use (Skills). In a few cases, lack of access to, or lack of knowledge of how to access, a validated tool such as the MMSE were the reasons cited for not using one (Environmental context and resources).

#### Category 3. GP refers to an aged care assessment service (ACAS) for cognitive assessment

Lack of time or resources (Environmental context and resources) was cited as one of the main reasons for a GP referring a patient to an ACAS for a cognitive assessment. An ACAS assessment was often needed for other reasons (*e.g*., assessment for eligibility for other services such as assistance in the home, respite care, or placement in an aged care facility) and, as it was believed a cognitive assessment formed part of this process, it was expedient for the GP to refer for a general ACAS assessment and not conduct a cognitive assessment himself (Beliefs about consequences): ‘To do a cognitive assessment, I think we do as an informal thing. When we believe that there’s a problem then … that’s where the memory clinic or the aged care assessment team come in’ (GP 02 – Rural).

Other reasons cited for having the cognitive assessment conducted by someone else was a preference for having it administered by an independent third party who would not influence the result (Beliefs about consequences). One GP saw it as providing an unbiased assessment: ‘Yeah we do [a cognitive assessment]. Although what I try to do is get someone else to do it for me so I’m not biased’ (GP 09 - Rural).

Another reason for the GP referring elsewhere to have the cognitive assessment carried out was a belief that it was uncomfortable for the patient to have their GP conduct it (Beliefs about consequences; Social influences). Some GPs may have preferred someone else to undertake the assessment because they themselves were uncomfortable conducting the assessment with their patients owing to the embarrassment it caused for both parties (Emotion), possibly due to lack of skills and confidence (Skills; Beliefs about capabilities).

#### Category 4. GP refers to a specialist for assessment without conducting a cognitive assessment

The reported reasons for referral to a specialist for assessment without conducting a cognitive assessment were similar to the reasons for referring to ACAS, particularly in terms of preferring to have a third party conduct the assessment, and patient/GP discomfort (Beliefs about consequences; Social influences; Emotion; Skills; Beliefs about capabilities). The main difference was that in some cases the referral occurred because the patient or carer had requested a specialist assessment and the GP was complying with their wishes (Social influences). The GP believed that there was no need for them to conduct a cognitive assessment in this case as the results would not affect the referral decision and the specialist would conduct a formal cognitive assessment anyway. As one GP stated: ‘No, I don’t do that on everybody. … If it’s clear that the family members want a referral to the other place, it won’t change my treatment and my referral process’ (GP 03 - Rural).

In some instances a formal cognitive assessment was considered unnecessary when the cognitive impairment appeared obvious (Beliefs about consequences): ‘And sometimes, you know, I don’t need Mini Mental when it’s clearly the problem. Then I will generally refer them on to the geriatrician, or psycho-geriatrician …’ (GP 21 – Metro).

#### Category 5. No cognitive assessment conducted by GP

In situations where no cognitive assessment was conducted, the main reason reported by participants was patient or carer refusal (Social influences). The other reason cited for not conducting a cognitive assessment was that the patient’s actual or suspected cognitive impairment wasn’t considered as problematic at the time, either by the GP, patient or carer, such that the GP decided it didn’t warrant further investigation by way of cognitive assessment (Beliefs about consequences). A patient’s other illnesses or life expectancy may also be taken into consideration (Beliefs about consequences). One GP believed that patients were able to self-report cognitive impairment without the need to conduct a formal cognitive assessment (Beliefs about consequences).

No differences were found between metropolitan and regional/rural GPs in terms of their reported practice and key influencing factors in relation to the conduct of cognitive assessments for patients with suspected cognitive impairment or dementia.

### Assessing co-morbid depression using a validated tool (recommended behaviour 2)

**Figure 2:**
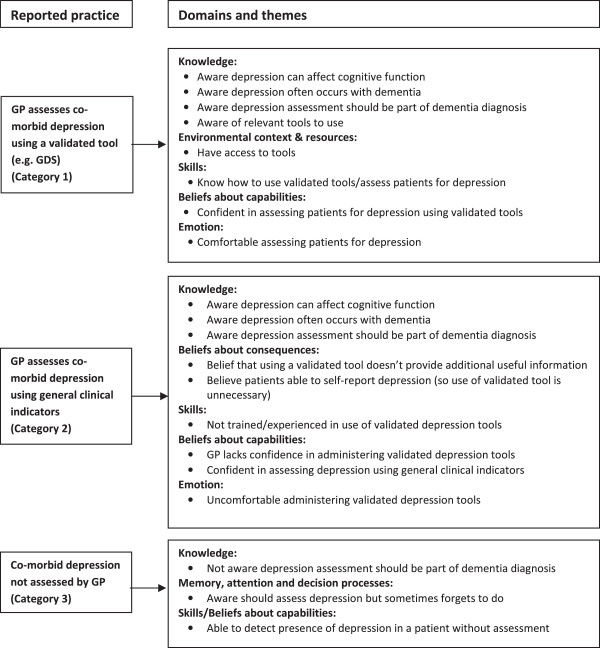
**Nature of reported practice and factors perceived to influence practice for recommended behaviour 2: assess co-morbid depression using a validated tool (*e.g *., GDS).**

A range of clinical behaviours engaged in by GPs relating to the assessment of co-morbid depression in patients with suspected cognitive impairment were identified. These were classified into three categories (Figure 2):

Category 1. GP assesses co-morbid depression using a validated tool.

Category 2. GP assesses co-morbid depression using general clinical indicators.

Category 3. Co-morbid depression not assessed by GP.

Unlike the situation with cognitive assessment, the GPs usually did not refer a patient elsewhere specifically for assessment of co-morbid depression, although this may have occurred by default when a patient was referred to ACAS or a specialist for a cognitive assessment.

Awareness that assessment for co-morbid depression should form part of the dementia diagnosis process was not as high amongst GPs as was their awareness of the need to conduct a cognitive assessment. While many GPs reported undertaking some form of depression assessment, in most situations they preferred to assess depression through general clinical indicators (Category 2) rather than by using a validated tool (Category 1). In a large number of cases, the GPs reported that no assessment of co-morbid depression was undertaken. The factors perceived to influence practice for each category are described below and summarised in Figure 2.

### Factors perceived to influence practice, mapped to the TDF

#### Categories 1 and 2. GP assesses co-morbid depression

The main factors influencing whether a patient with suspected cognitive impairment was assessed for co-morbid depression were awareness by the GP that depression could affect cognitive function, that depression often occurs with dementia, and that a depression assessment should form part of the dementia diagnosis process (Knowledge), as summed up by the following comment: ‘… depression can cause … poor concentration and memory impairment and, you know, sitting around not wanting to do anything and … all of those sort of things, so you’d really have to exclude that or … make some sort of assessment for that as well’ (GP 14 – Rural).

Those GPs who used general clinical indicators to assess for co-morbid depression rather than using a validated depression scale (Category 2) reported that validated scales provided no additional useful diagnostic information and as such offered no advantage over their general clinical assessment (Beliefs about consequences). This is illustrated in the following comments: ‘No, but depression is depression. In fact most of the time you don’t need a specific, you know, depression scale to diagnose the patient. So we do it clinically’ (GP 21 – Metro); ‘Oh, look, I was trying to use those so-called tools… but I found them no better than my intuition’ (GP 09 – Rural). Lack of experience or training in the use of validated scales possibly contributed to the preference for general clinical assessment (Skills). For example:

‘Probably through lack of training in the use of them. … I mean, if I was brought up using them, and experienced positive benefits from them, then I may be more willing to use them. .... But I have no experience with them in training’ (GP 03 – Rural).

This lack of experience or training may also have contributed to the discomfort expressed by some clinicians when administering a validated depression scale (Emotion; Beliefs about capabilities). A belief that patients were able to self-report depression, making use of a validated scale unnecessary, was another reason cited for not using such a tool (Beliefs about consequences).

Those who conducted a depression assessment using a validated tool were aware of the tests to use and had access to them (Knowledge; Environmental context and resources), were trained or experienced in using the tools (Skills), and were confident and comfortable administering them (Beliefs about capabilities; Emotion).

#### Category 3. Co-morbid depression not assessed by GP

The most frequently offered reason for not assessing co-morbid depression was that the GP was not aware that a depression assessment should form part of the diagnosis of dementia (Knowledge). Some GPs believed a depression assessment only needs to be conducted with patients in whom depression is suspected rather than being a routine part of the diagnosis process for all patients with suspected dementia (Knowledge). In some instances, for other GPs, a depression assessment may not be conducted because the clinician, although being aware of the need for it, forgets to do so (Memory, attention and decision processes).

In cases where the GP referred a patient to ACAS or a specialist without assessing their cognitive function they were also unlikely to assess the patient for co-morbid depression.

No differences were found between metropolitan and regional/rural GPs in terms of their practice and key influencing factors relating to assessment of co-morbid depression in patients with suspected dementia.

An overview of the influencing factors relating to the other recommended behaviours investigated in this study is contained in Additional file 2.

## Discussion

This research has explored the reported practice and theoretical explanations for why GPs may or may not practice in a manner consistent with recommended behaviours for the diagnosis and management of dementia. While the majority of GPs reported conducting a formal cognitive assessment using a validated scale, few GPs reported assessing co-morbid depression using a validated tool consistent with recommended guidelines. These findings are consistent with research suggesting that assessment of cognitive function by use of a MMSE is one of the first steps GPs take with patients when they suspect cognitive impairment [14,44] but that GPs in general prefer to use general clinical indicators rather than use of validated tools if they assess patients for co-morbid depression [45,46].

While previous studies have investigated barriers to the diagnosis and management of patients with dementia in primary care settings in Europe and North America [22,47-57], few studies have examined barriers to specific clinical behaviours, such as conduct of formal cognitive or depression assessments using validated tools [48,49,51,55,57], and no other study to our knowledge has reported an investigation of the barriers and enablers using a theoretical approach to guide data collection and analysis.

Awareness of the need to undertake a formal cognitive assessment using a validated scale (Knowledge), having the necessary skills to do so (Skills; Beliefs about capabilities), and time and resource availability (Environmental context and resources) were the main factors enabling conduct of formal cognitive assessment using a validated scale by GPs in this study. Most GPs reported conducting this assessment as part of their normal routine for evaluating patients in whom they suspect cognitive impairment or dementia (Behavioural regulation). This is consistent with previous findings in which US primary care physicians have reported conducting formal cognitive tests as part of their standard assessment procedures [48]. In the present study, key barriers to using a validated tool to conduct a cognitive assessment related to negative beliefs about the effect of using such a tool on patients (Beliefs about consequences), perceived limitations of the MMSE tool itself (Beliefs about consequences), and GP discomfort in administering it (Emotion). A reluctance to use formal cognitive tests because of concerns about insulting or embarrassing patients, the belief that such tests provide no additional information, and GP discomfort in administering these tests are consistent with previous reports [49,51,55,57]. Other barriers to formal cognitive assessment identified in other studies consistent with our findings include the time constraints in normal consultations and lack of GP skills in the use of formal cognitive tests [51,57,58]. However, new factors not previously reported were identified in this study. These included, for example, GPs’ referral to specialists, directly or through ACAS, for cognitive assessment and their reasons for doing so (*e.g*., lack of time/resources to conduct MMSE; limited confidence and skills in administering the MMSE themselves; being uncomfortable testing patients themselves; belief that a specialist would provide an unbiased and independent assessment). Also, factors influencing GPs’ failure to conduct themselves, or refer for, cognitive assessment were identified (*e.g*., beliefs that cognitive impairment was not sufficiently problematic so assessment perceived as unhelpful; refusal by patient or family; and GPs lacking skills to convince the patient and/or family of the need for assessment).

A key barrier to assessing co-morbid depression using a validated tool identified in this study was the belief that depression can be adequately assessed using general clinical indicators. Other barriers included: that the use of validated tools provided no additional useful information (Beliefs about consequences); discomfort in using validated tools (Emotion), possibly due to limited training, experience or confidence in using them (Skills; Beliefs about capabilities); limited awareness of the need for depression assessment (Knowledge); and forgetting to conduct the assessment (Memory, attention and decision processes). No previous studies to our knowledge have identified barriers to this recommended clinical behaviour, so these findings were important for informing our targeted practice change strategy.

Unlike many previous studies using the TDF to explore possible explanations for implementation problems, both content and thematic analyses were used in this study to analyse the interview data rather than use of a single approach [31,34,36,37,39,59]. First, the pattern of clinical behaviours that GPs engaged in, relating to each guideline recommendation, was identified and categorised using content analysis. For example, five categories of clinical behaviour were identified in relation to the recommendation that a formal cognitive assessment using a validated scale be conducted with individuals with suspected cognitive impairment. Next, content analysis of data within each category was conducted to identify the factors (*i.e*., barriers and enablers) perceived to influence the achievement of the recommended behaviour on the basis of identified categories (*e.g*., in instances where GPs report conducting no assessment of cognitive function, or in instances where GPs report conducting a cognitive assessment using a method or use of clinical criteria rather than a validated scale). Finally, the identified factors within each category were thematically mapped to the domains of the TDF. Thus our analysis drew upon both inductive ‘bottom up’ and deductive ‘top down’ approaches (the latter informed by the TDF) and enabled exploration of the theoretical explanations for why GPs may or may not practice in a manner consistent with recommended behaviours, detailed at the level of the identified categories of clinician behaviour (*e.g*., see Figure 1). While we found this approach useful in informing the design of our implementation intervention, further investigation is needed to determine the optimal approach to analysis when using the TDF to identify theoretical explanations for implementation problems and when designing implementation interventions to support practice change.

While this study has identified factors that may influence practice for managing patients with suspected cognitive impairment and dementia, there were some limitations. First, we invited a total of 700 GPs but conducted interviews with only 30 participants (4%) to achieve data saturation. The low response rate to our invitations may have been due to some individuals in the sampling frame being ineligible (*i.e*., not currently managing patients with suspected cognitive impairment or dementia) or possibly due to a perception that the clinical issue was of low priority given the diverse range of clinical problems that GPs manage given the multiple demands on GP time. However, despite this low response rate, participants varied on a range of variables including sex, years of experience, practice size, experience in caring for patients with dementia and geographical location, and saturation of themes was reached suggesting the findings are comprehensive in their coverage of the issues. Second, data presented in this study represent the perceptions and accounts of participants interviewed and, as such, may not represent actual influences on practice. Finally, only 20% of the interviews were coded in duplicate. A strength of the study was that researchers achieved a high level of consistency in terms of the barriers and enablers coded as being relevant.

Findings from this paper have been used to develop a targeted, theory-informed implementation intervention to support evidence-informed diagnosis and management of dementia by GPs. Using a theoretical approach enables a more comprehensive assessment of factors influencing practice and informs the matching of intervention components and behaviour change techniques to identified factors, and assists in conceptualising the pathway of change needed for the intervention to work [26,60]. An intervention informed by this study is being tested in a cluster randomised trial, the IRIS trial [40].

## Conclusions

To our knowledge, this is the first study to use a theoretical approach to investigate the barriers and enablers to guideline-recommended diagnosis and management of dementia in general practice. It has identified key factors likely to influence uptake of evidence-based dementia guidelines in Australian general practice. The results have been used to inform the development of a targeted, theory-informed implementation intervention aimed at supporting practice change in line with best evidence.

## Abbreviations

TDF: Theoretical domains framework; GP: General practitioner; MMSE: Mini-Mental state examination; IRIS: Investigating research implementation strategies for the care of elderly people with suspected cognitive impairment; SIGN: Scottish intercollegiate guidelines network; NICE: National Institute for Health and Clinical Excellence; SCIE: Social Care Institute for Excellence; AMPCo: Australian Medical Publishing Co; GDS: Geriatric depression scale; RACGP: Royal Australian College of General Practitioners; ACRRM: Australian College of Rural and Remote Medicine; ACAS: Aged care assessment service.

## Competing interests

MPE is joint founding editor of Implementation Science. DAO and SM are associate editors of Implementation Science. All editorial decisions regarding this manuscript were made independently by another editor. The remaining authors declare that they have no competing interests.

## Authors’ contributions

SEG, CJB, BW, LF, DAO, SDF and other members of the IRIS Study Group (Claire Harris [CH], Joanne E McKenzie [JEM], Duncan S Mortimer [DSM]) conceptualized and secured funding for the IRIS study, of which this qualitative study was one part. SEG was the lead investigator of the funding application. DAO, SEG, SM, JJF and KM designed this study. KM collected and analysed the data. KM, DAO, JJF and SM contributed to the interpretation of the data. KM wrote the first draft of the manuscript. DAO critically revised the manuscript in response to feedback. All authors contributed to manuscript refinement and take public responsibility for its content. All authors read and approved the final manuscript.

## Supplementary Material

Additional file 1 **Interview guide.** This file includes the interview guide used for GP interviews. Click here for file

Additional file 2 **Barriers and enablers to other recommended behaviours elicited in interviews.** This file includes the barriers and enablers to other recommended behaviours elicited in interviews. Click here for file
