# Supplementary material for: Understanding diagnosis and management of dementia and guideline implementation in general practice: a qualitative study using the theoretical domains framework
Source: Implement Sci. 2014 Mar 3;9:31. doi: 10.1186/1748-5908-9-31 (PMC4015883; doi:10.1186/1748-5908-9-31)
Supplement: Additional file 1 — Interview guide. This file includes the interview guide used for GP interviews. [file 1748-5908-9-31-S1.pdf]

Additional file 1. Interview guide

INTERVIEW GUIDE FOR GPS

IRIS
Investigating Research Implementation Strategies: care of people with suspected cognitive impairment or dementia

SEMI STRUCTURED INTERVIEW SCHEDULE

Introduction
As outlined in the explanatory statement, this study aims to identify and explore the factors that influence how you care for older patients with suspected cognitive impairment or dementia.

You do not have to answer every question and can cease the interview at any time. If you need to attend to an urgent matter we can stop the interview and recommence it later.

Firstly we will talk about how you become aware of patients with suspected cognitive impairment and what you usually do in these situations. Then we will talk about what you may do next to manage these patients and communicate with their carers, if they have one.

Before I start do you have any questions you would like to ask me?

Demographic / Background Information

- How many older patients do you currently have in your care with **suspected** cognitive impairment (living in the community, not in a nursing home)?
- How many of those with suspected cognitive impairment have come to your attention in the last 12 months?
- How many patients do you currently have in your care that have had a **confirmed** diagnosed of dementia (living in the community, not in a nursing home)?
- How many of those have been **diagnosed** in the last 12 months?

IDENTIFICATION OF SUSPECTED COGNITIVE IMPAIRMENT

I'm going to ask you to think of a recent patient you suspected had cognitive impairment.
- How did you become aware that the patient was experiencing cognitive changes? *(GP/patient/carer/other)*
- What were the factors that alerted X *(GP/patient/carer/other)* to the possibility of cognitive impairment?
- Over what period of time were these noticed?

Scenario 1: If raised by someone other than GP
- Had you noticed any of these signs/changes in the patient prior to the issue being raised by X?

Optional: If not mentioned
- Have you ever had a situation where you became concerned that a patient may have cognitive impairment and raised it with them or their carer?

If yes:
- Can you describe the situation and what occurred?

If no:
- How easy/difficult would it be to recognise possible cognitive impairment in a patient?
- What factors would influence this? Explore. Eg frequency see patients, familiarity with patient
- Can you envisage such a situation occurring in this practice/with your patients? Explore. Why or why not? What would alert you?
- Can you describe what you would do in this situation?
    - How would they raise the issue? With whom? How would they phrase it?

Scenario 2: If GP initiated through routine screening
- When is the screening conducted? Who is screened?

- What does it involve?
- How often do you detect someone with cognitive impairment through screening?

DIAGNOSIS

Recommended behaviours:

(i) Cognitive assessment if suspected cognitive impairment (MMSE)
(ii) Consider co-morbid depression and exclude as appropriate (by screening for depression)
(iii) Structural imaging
(iv) Medication review

What was the next step once you became aware of the situation (in the most recent patient diagnosed with dementia)?

Scenario 1: GP conducted assessment

- What did the assessment involve?
- Why did you choose/undertake/refer for (in case of imaging & pathology) those particular tests?
    - Explore use of cognitive tests and which test
    - Explore consideration of co-morbid depression and depression assessment; how done?
        - *If don't use a validated tool ask* – How assess? What questions ask? Why those questions?
    - Explore referral for structural imaging
    - Explore medication review. (What does it involve?)
  - How soon after becoming aware of the suspected cognitive impairment was the assessment undertaken?

Cover following domains (see possible prompt questions below if needed):
Beliefs about capabilities
Skills
Beliefs about consequences
Motivation/goals/behavioural regulation
Memory, attention, decisions
Environment
Social influences
Emotion
Professional role
Knowledge

Beliefs about capabilities

- What, if any, difficulties do you have in completing [each of] these assessments?
- Do you find doing a cognitive assessment easy or difficult to do? Explore *(if difficult what makes it difficult, if not difficult why?)*
- What about screening for depression? If difficult, what makes it difficult?
- Do you find reviewing the patient's medication easy or difficult to do? Explore (*if difficult what makes it difficult, if not difficult why?)*

Skills

- *What skills/training do you need to be able to do (each of) these assessments/review?*

Beliefs about consequences

- What are the benefits/advantages of conducting a cognitive assessment such as the MMSE?
    - And the disadvantages of not conducting it?
- What are the benefits/advantages of considering the presence of co-morbid depression and conducting a depression screen e.g. Geriatric Depression Scale (GDS)?
    - And the disadvantages of not screening for it?
- What are the benefits/advantages of referring for structural imaging?
    - And the disadvantages of not doing it?
- What are the benefits/advantages of reviewing the patient's medication?
    - And the disadvantages of not doing it?

Motivation/goals/behavioural regulation

- Why do you do [each of] these tests?
- Do you feel you should be doing these tests?

- Is this part of a routine you have for assessing patients with suspected cognitive dementia?

Memory, attention, decisions
- Is conducting each of these tests something you would routinely do in this situation or do you make a deliberate decision about each one?
- Would you ever forget to do one of the tests?

Environment
- To what extent do resources (e.g. time available, access to imaging & pathology facilities, ability to refer to specialists if needed, patient/carer co-operation) influence whether you do these tests?

Social influences
- In what way does the patient/carer/family influence your decision about conducting an assessment on a patient?
- Do you ever seek the opinion of colleagues before you make a decision about conducting these assessments on a patient?

Emotion
- Is conducting these assessments a difficult situation to deal with? Is it something you would prefer to avoid?

Professional role
- Do you think it is an appropriate part of your role to undertake each of these assessments? (i.e. cognitive assessment/ depression screen/ refer for structural imaging, review medication) or do you think another healthcare provider should be responsible for this?

- Are there any situations in which you wouldn't consider conducting the assessment yourself? Explore.

Scenario 2: GP referred elsewhere for assessment /diagnosis
- Who was the patient referred to?
- What were the reasons for referring to ----- (above)?
  (prompt for reasons e.g. skills, professional role, beliefs about capabilities, beliefs about consequences, resources, motivation/goals/behavioural regulation, environment, emotion etc)
- Which tests did you expect to be administered? *(cognitive assessment, depression assessment, structural imaging, pathology, medication review).Why those tests?*
- Did you consider conducting the assessment yourself? Why/why not? Explore. *Cover off domains e.g. skills, professional role, beliefs about consequences, resources, motivation/goals, emotion, social influences*
- How soon after becoming aware of the suspected cognitive impairment was the patient referred for assessment?
- Are there any situations in which you would consider conducting the assessment yourself? Explore.
- Who arranged the appointment? *GP, patient, carer, other?*
- How was the referral/patient followed up? Were you advised of the results of the assessment? What was the recommendation? Would you always be advised of the results in this situation?
- In what situations would referral differ?

Cover following domains (see possible prompt questions below if needed):
Memory, attention, decisions
Skills and Knowledge questions
Beliefs about capabilities
Professional role
Beliefs about consequences
Environment
Motivation
Social influences
Emotion?

Memory, attention, decisions
- What are your reasons for not doing the assessment/these tests yourself?
- Is referring to someone else to conduct the assessment something you would routinely do in this situation or do you deliberate about it with each patient?

Skills and Knowledge
- Are you familiar with how to complete a cognitive assessment (MMSE/others)?

- Are you familiar with the need to consider the presence of co-morbid depression as part of the diagnostic work-up of patients with suspected cognitive impairment?
- Do you know how to assess for depression?
- Are you familiar with the use of structural imaging (contrast CT or MRI) as part of the diagnostic work-up of patients with suspected cognitive impairment?
- Are you familiar with the need to review the patient's medication as part of the diagnostic work-up of patients with suspected cognitive impairment?

Beliefs about capabilities

- What, if any, difficulties do you have/have you had in completing [each of] these assessments?
  - Do you find doing a cognitive assessment easy or difficult to do? Explore *(if difficult what makes it difficult, if not difficult why?)*
  - What about assessing for depression? *(If difficult, what makes it difficult, if not why?)*
  - Do you find reviewing the patient's medication easy or difficult to do? Explore (*if difficult what makes it difficult, if not difficult why?)*

Professional role

- Do you think it is an appropriate part of your role to undertake each of these assessments? (i.e. cognitive assessment / depression assessment / refer for structural imaging / review medication) or do you think another healthcare provider should be responsible for this?

Beliefs about consequences

- What are the benefits/advantages of referring elsewhere for a cognitive assessment to be done?
  - And the disadvantages if you did conduct it?
- What are the benefits/advantages of referring elsewhere for a depression assessment to be conducted?
  - And the disadvantages if you did conduct it?
- What are the benefits/advantages of leaving (whoever refers to) to arrange the structural imaging?
  - And the disadvantages if you did arrange it?
- What are the benefits/advantages of leaving (whoever refers to) to review the patient's medication?
  - And the disadvantages if you did do it?

Motivation/goals/behavioural regulation

- Why don't you do this assessment?
- Is doing it something you want to do or you think someone else should do it?
- Is it a policy of this practice that this should be done by someone else?

Environment

- To what extent do resources (e.g. time available, access to imaging facilities, ability to refer to specialists, patient/carer co-operation) influence whether you refer elsewhere for assessment?

Social influences

- In what way does the patient/carer/family influence your decision about referring a patient elsewhere for assessment?
- Do you ever seek the opinion of colleagues before you make a decision about referring a patient?

Emotion

- Is this a difficult situation to deal with?
- Does referring to someone else to do the assessment make the situation easier for you?

<u>Scenario 3: No assessment conducted</u>

- Has a situation arisen where the decision was made not to conduct any assessment on a patient suspected of having dementia/cognitive impairment?
  - What were the circumstances? *e.g. patient/carer refused*
  - Why was the decision made? Explore. *Consider domains e.g. skills, resources, professional role, social influences, etc*
  - Who made the decision?
  - How did you/the patient/the carer feel about this decision?
  - How was this patient followed up/managed?
  - Is this situation common?

After covered domains ask:

What would your normal procedure be in other situations (where patient/carer agrees to assessment being conducted?

Explore with questions for Scenario 1 or Scenario 2 above.

Cover following domains (see possible prompt questions below if needed):
Memory, attention, decisions
Motivation/goals/behavioural regulation
Professional role
Skills and Knowledge
Beliefs about capabilities
Beliefs about consequences
Environment
Social influences
Emotion

Memory, attention, decisions

- What are/were your reasons for not doing the assessment/these tests?
- Was it a deliberate decision not to do it in this situation (or was it something you forgot to do)?

Motivation/goals/behavioural regulation

- Why didn't you do the assessment?
- Do you feel you should be doing it?
- Is it part of a routine you have for patients with suspected cognitive dementia?

Professional role

- Do you think it is an appropriate part of your role to undertake each of these assessments? (i.e. cognitive assessments/depression assessment/ refer for structural imaging/pathology, review medication) or do you think another healthcare provider should be responsible for this?

Skills and Knowledge)

- Are you familiar with how to complete a cognitive assessment (MMSE/others)?
- Are you familiar with the need to consider the presence of co-morbid depression as part of the diagnostic work-up of patients with suspected cognitive impairment?
- Do you know how to assess for depression?
- Are you familiar with the use of structural imaging (contrast CT or MRI) as part of the diagnostic work-up of patients with suspected cognitive impairment?
- Are you familiar with the need to review the patient's medication as part of the diagnostic work-up of patients with suspected cognitive impairment?

Beliefs about capabilities

- What, if any, difficulties do you have/have you had in completing [each of] these assessments?
    - Do you find doing a cognitive assessment easy or difficult to do? Explore *(if difficult what makes it difficult, if not difficult why?)*
    - What about assessing for depression? *(If difficult, what makes it difficult, if not difficult why?)*
    - Do you find reviewing the patient's medication easy or difficult to do? Explore (*if difficult what makes it difficult, if not difficult why?)*

Beliefs about consequences

- What would the benefits/advantages be if you did/could conduct these assessments (cognitive assessment, depression assessment, refer for structural imaging, review the patient's medication)?
- And the disadvantages/implications if you don't/can't conduct it?

Environment

- To what extent do resources (e.g. time available, access to imaging & pathology facilities, ability to refer to specialists if needed, patient/carer co-operation) influence whether you do these tests?

Social influences

- In what way does the patient/carer/family influence your decision about conducting an assessment?
- Do you ever seek the opinion of colleagues before you make a decision about conducting an assessment?

Emotion

- If you don't/can't refer a patient for assessment does this leave you feeling uncomfortable about the situation? (Explore in what way).
- Do you have any concerns about their clinical condition in this situation?

DIAGNOSIS DISCLOSURE

Now I am going to ask you about a patient that has been **diagnosed** with dementia (*ICD-10 definition on sheet for interviewer*). (Emphasise that now talking about a **diagnosed** patient not a patient with suspected dementia).

What would the normal timeframe be from when a patient is first identified as possibly having dementia to actually being diagnosed?

- o How long does the assessment process usually take?

How is a patient informed about their diagnosis?
Who informs them?
If GP, how do you tell them? What do you say/what words do you use?
At what stage do you tell them? (eg when suspect they have dementia or when definitively diagnosed).
Are patients always told of the diagnosis?
Are carers/family members always told of the diagnosis?
Who decides who will be told?

MANAGEMENT

Recommended behaviours:

(i) Referral to a specialist for dementia modifying medications
(ii) Recreational activities introduced to people with dementia
(iii) Cognitive stimulation offered to people with dementia
(iv) Caregiver training on effective interventions for people with dementia

Now I would like to talk to you about the management and follow-up of patients who have been diagnosed with dementia.

- Who is (primarily) responsible for the ongoing management of the patient? (e.g. GP, specialist, other).
- Who else is involved? (e.g. specialist, CDAMS, ACAS, other) What does the ongoing management of the patient involve?

Medication management

Referral to a specialist for dementia modifying medication
(If not already raised)

- Do you offer patients the opportunity to be referred to a specialist with a view to being prescribed dementia modifying medication?
- How do patients/carers react?
- Who do you refer them to? Why?
- In which situations wouldn't you offer this to a patient?
- Who is responsible for the ongoing management of this medication?

Cover following domains (see possible prompt questions below if needed):
Skills and Knowledge
Memory, attention, decisions
Motivation
Professional role
Beliefs about capabilities
Beliefs about consequences
Environment
Social influences
Emotion?

Skills & Knowledge

- Are you familiar with/or know about the dementia modifying medications that are available?
- Are you familiar with/or know about the regulations regarding the administration of these medications?

Memory, attention, decisions

- Is referring patients to a specialist for this medication something you would do with all patient or do you consider the individual situation?
- If you don't refer them for this what are your reasons?

Motivation/goals/behavioural regulation
- Do you feel you should be referring patients for this medication?
- Is it part of your routine you have with patients in this situation?

Professional role
- Do you think it is an appropriate part of your job/role to refer patients for these medications?

Beliefs about capabilities
- What, if any, difficulties do you have/have you had in referring patients for these medications? Explore *(If difficult, what makes it difficult, if not difficult why?)*

Beliefs about consequences
- What are the benefits/advantages of patients being referred for these medications?
    - And the disadvantages of them not being referred?

Environment
- To what extent do resources (eg time available, access to specialists, patient/carer co-operation) influence whether you refer patients for these medications?

Social influences
- In what way does the patient/carer/family influence your decision about referring patients for this medication?
- Do you ever seek the opinion of colleagues before you make a decision about referring a patient for this medication?

Emotion
- Is this a difficult situation to deal with? Is it something you would prefer to avoid?
- If you don't offer patients the opportunity to be referred to a specialist for dementia modifying medication, or the patient refuses, does that ever leave you feeling uncomfortable about their condition?

Non-medication Management (Psychosocial management)

- How are the non-medical/psychosocial aspects of dementia managed?
  (e.g. recreational activities, cognitive functioning/stimulation, caregiver training/support

Recreational activities.
- What do you recommend to patients in relation to recreational activities?
        - What advice do you provide? What do you suggest they do? (eg refer to AA program or recommend activities)
        - Why do you suggest this?
        - At what stage do you suggest this (eg when first diagnosed/later).

What, if any, follow-up is undertaken? How frequently?

Cognitive functioning/stimulation.
- What do you recommend to patients to assist with cognitive functioning?
    - What advice do you provide? What do you suggest they do? (eg refer to AA program or recommend relevant activities).
    - Why do you suggest this?
    - At what stage do you suggest this (eg when first diagnosed/later).
    - What, if any, follow-up is undertaken? How frequently?

Caregiver training/education.
- What is recommended to assist caregivers?
    - What advice do you provide to caregivers?
    - Do you recommend any training/education for caregivers? (eg AA program)
        - What does it involve?
        - Who provides it?
        - What are the reasons for recommending this?

- At what stage do you suggest this (eg when first diagnosed/later).
- What, if any, follow-up is undertaken?

Cover following domains (see possible prompt questions below if needed:
Skills and Knowledge
Memory, attention, decisions
Motivation
Professional role
Beliefs about capabilities
Beliefs about consequences
Environment
Social influences
Emotion

Skills & Knowledge

- Are you familiar with/or know what advice should be provided to patients/carers regarding cognitive stimulation? And recreational activities?
- Are you familiar with/or know what training/education is available for caregivers?

Memory, attention, decisions

- Is advising patients about cognitive stimulation/recreational activities something you would do with each patient or do you consider the individual situation?
- If you don't advise them about this what are your reasons:
    - Cognitive stimulation activities?
    - Recreational activities?
- Is advising carers about the training/education that is available something you would do with each carer or do you consider the individual situation?
- If you don't advise them about this what are your reasons?

Motivation/goals/behavioural regulation

- Do you feel you should be advising patients/carers about cognitive stimulation activities/recreational activities?
- Do you feel you should be advising cares about training/education that is available?
- Is it part of your routine you have with patients & carers in this situation?

Professional role

- Do you think it is an appropriate part of your role to recommend [each of] these activities or is this the role of someone else?

Beliefs about capabilities

- What, if any, difficulties do you have/have you had in advising patients about
    - Cognitive stimulation activities?
    - Recreational activities?
    - Explore *(if difficult what makes it difficult, if not difficult why?)*
- What, if any, difficulties do you have/have you had in advising carers about training/education programs?
    - Explore *(If difficult, what makes it difficult, if not difficult why?)*

Beliefs about consequences

- What are the benefits/advantages of patients being advised about cognitive stimulation activities?
    - And the disadvantages of them not being advised about this?
- What are the benefits/advantages of patients being advised about recreational activities?
    - And the disadvantages of them not being advised them about this?
- What are the benefits/advantages of caregivers being advised about training/education that is available?
    - And the disadvantages of them not being advised about this?

Environment

- To what extent do resources (eg time available, access to information/service providers, patient/carer co-operation) influence whether you advise patients/carers about:
    - Cognitive stimulation activities
    - Recreational activities
    - Training/education available for caregivers

Social influences

- In what way does the patient/carer/family influence your decision about advising patients about;
    - Cognitive stimulation activities
    - Recreational activities
    - Training/education available for caregivers

- Do you ever seek advice/information from colleagues/other service providers before you advise patients about:
    - Cognitive stimulation activities
    - Recreational activities
    - Training/education available for caregivers

Emotion
- Is this a difficult situation to deal with? Is it something you would prefer to avoid?

Clinical Practice Guidelines
- Are you aware of any Clinical Practice Guidelines in relation to the diagnosis of people with suspected cognitive impairment and management of people with dementia in general practice?
    - *If yes:* What is your understanding of the recommendations for diagnosis and management?
    - *If no:* The recommendations for diagnosis and management of people with dementia are

Particularly discuss targeted recommendations they are currently not doing.
Documents to refer to:
- Dementia Flowchart

Other (optional)
- Is there anything else about the diagnosis of people with suspected cognitive impairment and their ongoing management that you would like to mention that we haven't already covered?
- Is there anything/anyone that you have found to be particularly helpful in assisting you top manage patients with dementia/cognitive impairment?
- Who would you trust/consider to be a local opinion leader/expert in the diagnosis and management of people with dementia?

ADDITIONAL ISSUES TO BE EXPLORED (IF POSSIBLE)

(i) Referral for pathology tests
(ii) Driving assessment
(iii) Social/legal aspects of care
(iv) Advice re respite care

Do you refer patients for pathology tests as part of the initial assessment
- What is the reason for this (benefits/advantages)?
- And the disadvantages of not doing it?
- Which tests do you request (show checklist if necessary)?
- Why those tests?

Do you discuss the potential impact of dementia on driving safety/capability with the patient/carer? If not, explore reasons

Do you discuss any of the following with the patient/carer?

- Capacity to make legal decisions
- Capacity to consent to medical treatment
- Capacity to make a will

If not, explore reasons

Do you discuss with the carer the importance of taking a break/respite care?
- What information do you provide?
- Who do you refer them to?

How is this handled if the carer is not your patient?
