# Supplementary material for: Understanding diagnosis and management of dementia and guideline implementation in general practice: a qualitative study using the theoretical domains framework
Source: Implement Sci. 2014 Mar 3;9:31. doi: 10.1186/1748-5908-9-31 (PMC4015883; doi:10.1186/1748-5908-9-31)
Supplement: Additional file 2 — Barriers and enablers to other recommended behaviours elicited in interviews. This file includes the barriers and enablers to other recommended behaviours elicited in interviews. [file 1748-5908-9-31-S2.pdf]

Additional file 2. Barriers and enablers to other recommended behaviours elicited in interviews

Clinical behaviours	Barriers	Enablers
Refer for pathology testing	- None cited	- Believe pathology necessary to rule out other possible causes of dementia-like symptoms<br>- Pathology required to refer patient to specialist/CDAMS<br>- Pathology services easily accessible<br>- Pathology testing a routine procedure
Refer for head/brain CT scan	- Believe brain CT not necessary/helpful for all patients<br>- Believe brain CT not needed to refer patient to specialist/CDAMS<br>- Not aware brain CT should form part of the diagnostic work up of patients with suspected dementia<br>- Difficult for patients to access CT facilities	- Brain CT needed to refer patient to specialist/CDAMS<br>- Able to access CT facilities<br>- Believe brain CT useful/necessary to rule out other conditions<br>- Brain CT helps to define type of dementia
Review current medication	- Complicated medication regimen requires pharmacist review<br>- Doesn't believe review of medication is part of diagnostic work up of suspected dementia	- Aware some medications affect cognitive function<br>- Believe review of current medication is part of diagnostic work up of suspected dementia<br>- Reviewing medications is a routine procedure
Disclose or reinforce a diagnosis of dementia	- Uncomfortable discussing issue<br>- Not wish to upset the patient<br>- Believe labelling the disease not important<br>- Believe some patients find label confronting/stigmatising<br>- Believe some patients not able to understand diagnosis<br>- Believe more important for carer to know than patient	- Believe important to be honest with patients about their diagnosis<br>- Have a responsibility to patient to disclose the diagnosis<br>- Comfortable discussing issue<br>- Believe patient is aware of the diagnosis (due to consultation with specialist) so shouldn't avoid talking about it<br>- Believe patient (and carer) need to know so they can prepare for the future
Refer to specialist (including via CDAMS) for access to dementia-modifying medications	- Patient's other medical conditions considered a higher priority; patient too ill for other treatment<br>- Cognitive impairment level not considered sufficient to be eligible for medication<br>- GP believes the medication is not particularly effective<br>- Difficulty accessing CDAMS/specialist (for review and commencement of medication)<br>- Patient/carer refusal<br>- Refer only in instances where patients believed to have Alzheimer's dementia<br>- Formal diagnosis considered unnecessary and won't affect patients management	- Need for specialist authorisation to access medication<br>- Need for confirmation of diagnosis<br>- Desire to access support services provided/coordinated by CDAMS/specialist referral (e.g. care packages, respite, carer support)<br>- Able to access CDAMS/specialist<br>- Patient/carer request for specialist review

Provide information on, or refer for, recreational and activities to promote cognitive stimulation	- Believe this is done by others (e.g. CDAMS, ACAS, specialist recommends information or refers patients and carers)<br>- Believe patients/carer will organise<br>- Believe not all patients interested<br>- Consider some patients incapable of doing such activities<br>- Only when requested or believe needed<br>- Not aware of what available/appropriate<br>- Forget to do<br>- Believe patient already undertaking cognitive/recreational activities	- Routine management for patients with dementia and their carers<br>- Routine management for older patients<br>- Believe beneficial for patients and carers (effects for patients with dementia; respite for carer)<br>- Aware of programmes/activities available and appropriate
Provide, or refer for, caregiver training	- GP believes CDAMS/specialist organises or refers carer to appropriate place<br>- GP believes carer has organised/will organise if required<br>- Not aware that formal training programmes available<br>- Only recommends when requested by carer or believes needed<br>- Forgets/doesn't think to do<br>- GP believes many carers not interested<br>- Carer refusal	- GP aware of training programmes (e.g. Alzheimer's Australia)<br>- Believes programmes are beneficial
Promote awareness of changing driving capacity as disease progresses	- Believe inability to drive causes practical difficulties for patients, especially in country areas (e.g. shopping, accessing appointments) and emotional/social impacts (e.g. loss of independence, social isolation), so possible risk has to be weighed against these<br>- Considers it a difficult issue to deal with so avoids as long as possible<br>- Considers low risk if patient continues driving (esp. in early stages)<br>- Concerned about effect on doctor-patient relationship if GP seen to be involved<br>- Believe GPs shouldn't be responsible for dealing with this issue	- Important issue that needs to be dealt with<br>- Concern about the risk to patients and others<br>- Believe role of GP to deal with this issue<br>- While inability to drive may cause difficulties for patients, considers it more important to respond to changing driving capacity
Discuss legal issues	- Believe this is dealt with by others (e.g. CDAMS, ACAS)<br>- Considers it a difficult issue so avoids if possible<br>- Believe doesn't have the knowledge/skills/experience to deal with this issue<br>- Believe once dementia diagnosis made it is too late to deal with these issues; they need to be done beforehand	- Important issue that needs to be dealt with<br>- Believe will cause problems later for patient, carer and GP if not dealt with<br>- Believe it is GPs' role to discuss<br>- Refers to appropriate sources to organise if GP doesn't have sufficient knowledge/skills

	- Not think to raise issue<br>- Believe patients/carers usually deal with issue themselves	
